# Novel oral histone deacetylase inhibitor, MPT0E028, displays potent growth-inhibitory activity against human B-cell lymphoma *in vitro* and *in vivo*

**DOI:** 10.18632/oncotarget.3213

**Published:** 2014-12-31

**Authors:** Han-Li Huang, Chieh-Yu Peng, Mei-Jung Lai, Chun-Han Chen, Hsueh-Yun Lee, Jing-Chi Wang, Jing-Ping Liou, Shiow-Lin Pan, Che-Ming Teng

**Affiliations:** ^1^ Pharmacological Institute, College of Medicine, National Taiwan University, Taipei, Taiwan; ^2^ Chinese Medicine Research and Development Center, China Medical University Hospital, Taichung, Taiwan; ^3^ School of Pharmacy, China Medical University, Taichung, Taiwan; ^4^ Center for Translational Medicine, Taipei Medical University, Taipei, Taiwan; ^5^ School of Pharmacy, College of Pharmacy, Taipei Medical University, Taipei, Taiwan; ^6^ The Ph.D. Program for Cancer Biology and Drug Discovery, College of Medical Science and Technology, Taipei Medical University, Taipei, Taiwan

**Keywords:** MPT0E028, B-cell lymphoma, Histone deacetylase (HDAC), Akt, apoptosis

## Abstract

Histone deacetylase (HDAC) inhibitor has been a promising therapeutic option in cancer therapy due to its ability to induce growth arrest, differentiation, and apoptosis. In this study, we demonstrated that MPT0E028, a novel HDAC inhibitor, reduces the viability of B-cell lymphomas by inducing apoptosis and shows a more potent HDAC inhibitory effect compared to SAHA, the first HDAC inhibitor approved by the FDA. In addition to HDACs inhibition, MPT0E028 also possesses potent direct Akt targeting ability as measured by the kinome diversity screening assay. Also, MPT0E028 reduces Akt phosphorylation in B-cell lymphoma with an IC_50_ value lower than SAHA. Transient transfection assay revealed that both targeting HDACs and Akt contribute to the apoptosis induced by MPT0E028, with both mechanisms functioning independently. Microarray analysis also shows that MPT0E028 may regulate many oncogenes expression (e.g., TP53, MYC, STAT family). Furthermore, *in vivo* animal model experiments demonstrated that MPT0E028 (50–200 mg/kg, po, qd) prolongs the survival rate of mice bearing human B-cell lymphoma Ramos cells and inhibits tumor growth in BJAB xenograft model. In summary, MPT0E028 possesses strong *in vitro* and *in vivo* activity against malignant cells, representing a potential therapeutic approach for cancer therapy.

## INTRODUCTION

According to the American Cancer Society, B-cell lymphomas make up approximately 85 percent of non-Hodgkin lymphomas (NHLs) in the United States [1]. Current strategies for the treatment of NHLs include the use of monoclonal antibodies (e.g., rituximab), either as single agent or in combination with chemotherapy and radiotherapy. However, monoclonal antibodies often result in relapse and resistance due to a variety of molecular mechanisms. Therefore, discovery and development of novel anticancer agents to improve overall survival in cancer patients becomes an essential and urgent medical need. Target therapy, employing proteasome inhibitors, mTOR inhibitors, tyrosine kinase inhibitors, and histone deacetylase inhibitors (HDACi), plays an important role in clinical oncology [2], suggesting these pathways represent key signal transductions in lymphoma tumorigenesis.

Histone deacetylases (HDACs) are a family of enzymes that remove the acetyl group from histone lysine tails, leading to chromatin condensation and transcriptional repression. At present, there are four classes of HDACs: class I (HDAC1, 2, 3, and 8) mainly localizes in the nucleus; class II (IIa: HDAC4, 5, 7, and 9; IIb: HAC6, and 10) may shuttle between cytoplasm and nucleus. Class III HDACs, also known as sirtuins (sirtuins 1–7), requires NAD^+^ as a cofactor for enzymatic activity; however, HDAC11 is the sole member of HDAC11. Class I, II, and IV are classical HDACs which need Zn^2+^ for enzymatic activity and are inhibited by HDAC inhibitors that chelate Zn^2+^ ion in their catalytic sites. HDAC inhibitors have multiple mechanisms of inducing cell cycle arrest, cell differentiation, and cell death through apoptosis, autophagy, or necrosis in many cancer cells. They have also shown to inhibit angiogenesis, migration, and metastasis [3, 4]. Consequently, HDAC inhibitors, proven to be particularly effective against hematological malignancies in clinical trials, are increasingly perceived as promising anticancer agents [5]. Vorinostat [6] and Romidepsin [7] are two HDAC inhibitors recently approved by the U.S. Food and Drug Administration (FDA) for cutaneous T-cell lymphoma (CTCL).

Upregulation of the PI3K/Akt/mTOR pathway occurs in many human cancers, including lymphoma; therefore, this pathway is considered a target for anticancer therapy in several human cancer types [8]. Activation of phosphatidylinositol-3 kinase (PI3K) enables recruitment of the serine/threonine kinase Akt to the cell membrane and then phosphorylates and activates Akt. Akt then activates the downstream protein mammalian target of rapamycin (mTOR), which phosphorylates translation initiation through ribosomal p70S6 kinase (p70S6k) or eukaryotic translation initiation factor 4E (eIF4E) binding proteins (4E-BPs). This promotes dissociation from the translation factor eIF4E, which stimulates RNA translation. On the other hand, Akt could activate GSK3β to manipulate cell cycle and glucose metabolism [9]. Akt is also a key regulator promoting cell growth and cell survival [10], which dysregulated causes tumor progression in many cancers. Therefore, many Akt inhibitors are being developed for clinical investigation [11].

MPT0E028 (3-(1-Benzenesulfonyl-2,3-dihydro-1H-indol-5-yl)-N-hydroxy-acrylamide) is a novel HDAC inhibitor *in vitro* and *in vivo* with a potent and broad HDAC inhibitory effect in multiple human cancers, both alone and in combination with other treatments [12-14]. In this study, we show that MPT0E028 possesses a more potent inhibitory effect against HDACs and greater ability in targeting Akt compared with the HDAC inhibitor vorinostat (SAHA) in human B-cell lymphoma cells. In an *in vivo* study, MPT0E028 prolonged the survival rate of mice bearing human lymphoma Ramos cells and significantly suppressed human lymphoma BJAB tumor xenograft growth; using the same dose, the effect of SAHA was considerably weaker. According to our previous findings and encouraging results, MPT0E028 manifests potent preclinical activity against human B-cell lymphoma, making this HDAC inhibitor a promising agent for hematologic cancer treatment.

## RESULTS

### MPT0E028 induces apoptosis in B-cell lymphoma cells

**Figure 1:**
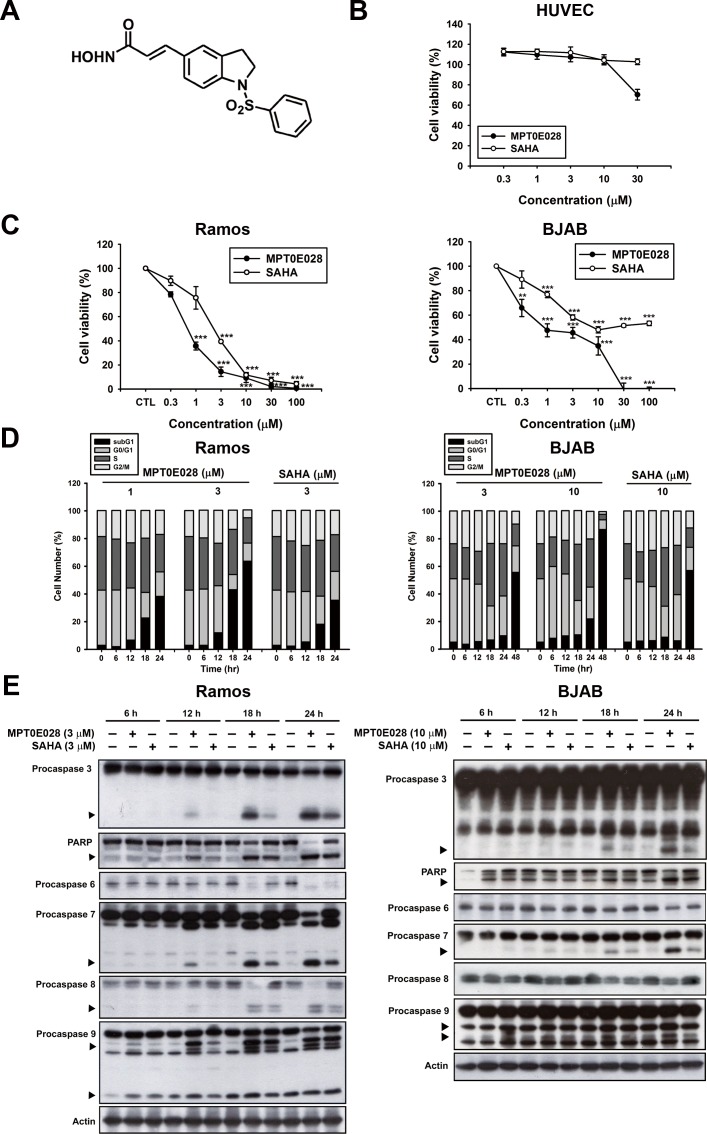
MPT0E028-induced apoptosis in human lymphoma cell lines A. structure of MPT0E028. B. Non-cytotoxic effect of MPT0E028 against normal cell line. C. Concentration-dependent effect of MPT0E028 or SAHA on the inhibition of cell growth in B-cell lymphoma cell lines. In B and C, HUVEC, Ramos, and BJAB cells were treated with different concentrations of MPT0E028 or SAHA for 24 h, and then cell viability was determined by MTT assay. Data represent mean ± SEM from at least three independent experiments (*P < 0.05; **P < 0.01; ***P < 0.001; compared with the respective control group). D. Time- and concentration-dependent effect of MPT0E028 and SAHA on the progression of subG1 population. Ramos and BJAB cells were treated with different concentrations of MPT0E028 or SAHA for the indicated times and then cell cycles were determined by flow cytometry. E. Effect of MPT0E028 and SAHA on caspases and PARP activation. Ramos and BJAB cells were treated with indicated concentration of MPT0E028 or SAHA for the indicated time, and then whole-cell lysates were subjected to western analysis detecting caspase 3, 6, 7, 8, 9, and PARP.

First, we assayed two human B-cell lymphoma cells, Ramos and BJAB, for viability and human normal HUVEC cells for toxicity in the presence of various concentrations of MPT0E028 and SAHA for comparison. MPT0E028 (Fig. 1A) showed no toxic effect on human normal HUVEC cells (IC_50_ > 30 μM) (Fig. 1B), but induced significant concentration-dependent growth inhibition both in Ramos (IC_50_ = 0.65 ± 0.1 μM) and BJAB lymphoma cells (IC_50_ = 1.45 ± 0.5 μM) compared with SAHA (IC_50_ = 2.61 ± 0.4 and 44.22 ± 10.0μM in Ramos and BJAB cells, respectively) (Fig. 1C). We also used FACS cytometry to analyze cell cycle progression and found that MPT0E028 substantially increased the subG1 phase population in a time- and concentration-dependent manner (Fig. 1D). Further, we used western blot analysis to characterize several caspases and PARP activation following the treatment of MPT0E028 at the indicated time. The results show that MPT0E028 induced caspase-3 and PARP cleavages, as well as caspase-6, -7, -8, and -9 activation in both cells (Fig. 1E). The data are consistent with that of flow cytometry, suggesting that MPT0E028 may induce apoptotic cell death. Taken together, MPT0E028 substantially induces growth inhibition and apoptosis more efficaciously than SAHA in a concentration- and time-dependent manner in human B-cell lymphoma cells.

### MPT0E028 inhibits histone deacetylase (HDAC) enzyme activity and induces apoptosis through HDAC inhibition

**Figure 2:**
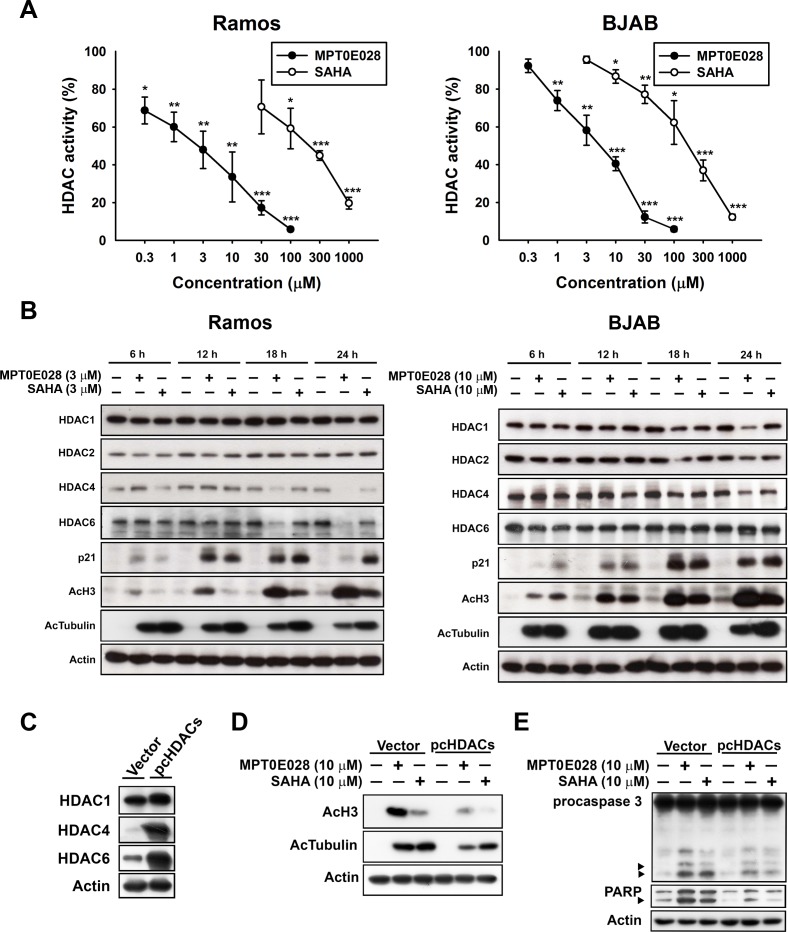
Effect of MPT0E028 on HDAC enzyme activity and protein expression in human B-cell lymphoma cells A. Effect of MPT0E028 on HDAC enzyme activity. Ramos and BJAB cells were treated with different concentrations of MPT0E028 or SAHA for 24 h and then HDAC activities were detected as described in Materials and Methods. Data represent mean ± SEM from at least three independent experiments. (*P < 0.05; **P < 0.01; ***P < 0.001; compared with the respective control group) B. Effect of MPT0E028 on HDAC protein expression and HDAC inhibition markers. Cells were treated with MPT0E028 or SAHA for the indicated time interval and then harvested for HDAC1, 2, 4, 6, and HDAC inhibition marker (acetyl-histone 3, acetyl-α-tubulin, and p21) detection using western blotting. C. Confirmation of the transfection effect of HDACs coexpression. D. Effect of HDACs coexpression on MPT0E028-induced HDAC inhibition markers upregulation. E. Contribution of HDACs to MPT0E028-induced cell apoptosis. BJAB cells were transiently transfected with plasmids encoding vector or flag-tagged human HDAC1, 4, and 6 by using nucleofection described in Materials and Methods. Whole cell lysates were subjected to western blotting for C. HDACs detection and then treated with 10 μM MPT0E028 or SAHA for 24 h. Whole cell lysates were subjected to western blotting for D. acetyl-histone 3, acetyl-α-tubulin. E. caspase 3, and PARP detection.

We have previously determined that MPT0E028 is a pan-HDAC inhibitor through direct HDAC targeting [12]. In our previous study, we used an enzyme-based HDAC fluorescence activity assay to detect five isoforms of HDACs: HDAC1, 2, and 8 from class I; HDAC4 from class IIa; and HDAC6 from class IIb. The results showed that MPT0E028 inhibited HDAC1, 2, 6, and 8 with an IC_50_ value in the nanomolar range (29.48–2532.57 nM) but had no effect upon HDAC4, representing a more potent HDAC inhibitory spectrum than SAHA [12]. In the present study, we assessed HDAC enzyme activity under treatment of MPT0E028 and SAHA in human B-cell lymphoma by using cell-based HDAC fluorescence activity assay. As shown in Fig. 2A, MPT0E028 effectively inhibited HDAC enzyme activity in a concentration-dependent manner (IC_50_ = 2.88 ± 1.9 μM in Ramos, 4.54 ± 1.2 μM in BJAB), whereas the inhibitory activity of SAHA was much weaker (IC_50_= 143.48 ± 67.4 μM in Ramos, 149.66 ± 40.3 μM in BJAB). Protein expression of HDAC1, 2, and 4 was also slightly downregulated after 24 h treatment of MPT0E028 in both cell lines, whereas HDAC6 was downregulated in Ramos cells but only slightly cleaved in BJAB cells, as determined by western blotting (Fig. 2B). Biomarkers of HDAC inhibition, which include hyperacetylation of histone 3, α-tubulin, and upregulation of p21 expression [15], were all detected under treatment of MPT0E028 in a time-dependent manner in both Ramos and BJAB cells (Fig. 2B). To determine whether inhibition of HDACs is involved in the apoptosis induced by MPT0E028, transient transfection was performed using flag-tagged pcHDAC1, 4, and 6 for HDAC coexpression and pcDNA3.1 vector as control. As shown in Fig. 2C, HDAC1, 4, and 6 were all transfected successfully, and the acetylation level of histone 3 and α-tubulin were reversed when HDACs were coexpressed (Fig. 2D). MPT0E028-induced caspase 3 and PARP activation were also abolished by coexpressing HDACs (Fig. 2E), suggesting that MPT0E028 induces human B-cell lymphoma cells apoptosis through inhibition of HDACs.

### MPT0E028 inhibits Akt/mTOR pathway activation

**Figure 3:**
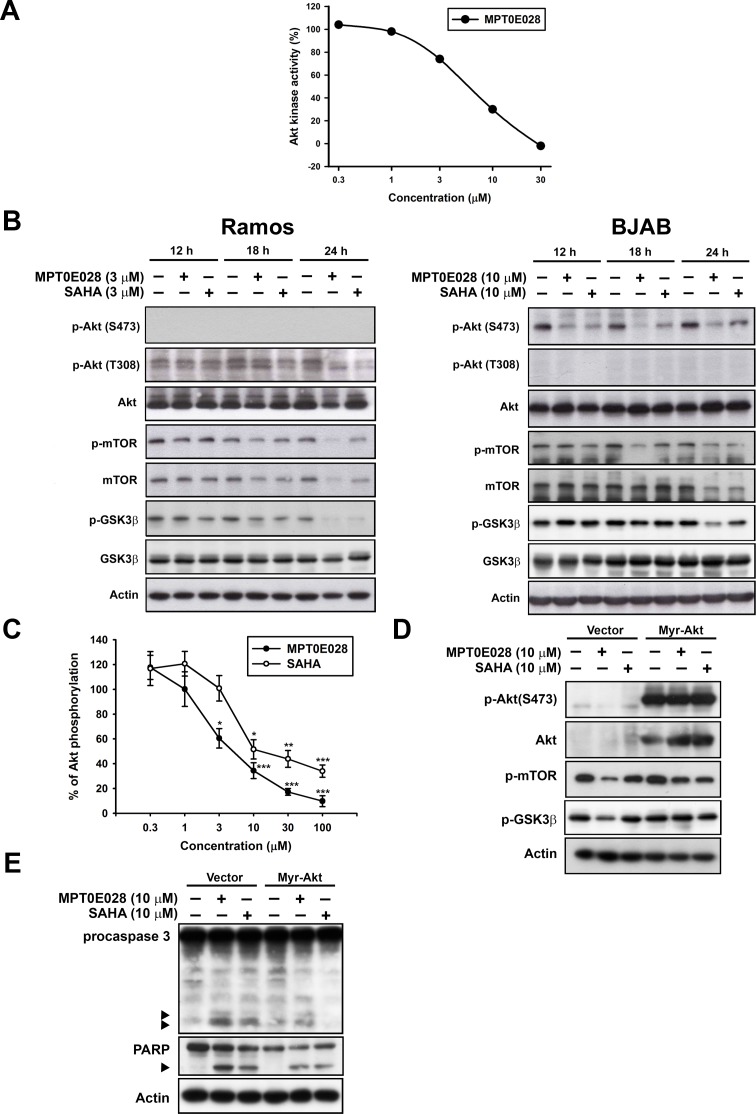
Effect of MPT0E028 on Akt and downstream proteins A. Effect of Akt kinase activity inhibited by MPT0E028. Detailed procedure is described in Materials and Methods. B. Effect of MPT0E028 on Akt and downstream proteins phosphorylation. Cells were treated with MPT0E028 or SAHA for the indicated time interval and then harvested for p-Akt (S473), p-Akt (T308), Akt, and Akt downstreram proteins (mTOR and GSK3β) detection using western blotting. C. Akt phosphorylation inhibited by MPT0E028. BJAB cells were treated with or without MPT0E028 or SAHA (0.3 – 100 μM) for 24 h and then harvested for p-Akt (S473) detection using Akt ELISA kit. Data represent mean ± SEM from at least three independent experiments. (*P < 0.05; **P < 0.01; ***P < 0.001; compared with the respective control group) D. Confirmation of the transfection effect of constitutive active myr-Akt and downstream proteins. E. Contribution of Akt to MPT0E028-induced cell apoptosis. In D and E, BJAB cells were transiently transfected with plasmids encoding vector or myr-Akt by using nucleofection described in Materials and Methods and treated with or without MPT0E028 or SAHA (10 μM) for 24 h. Whole cell lysates were subjected to western blotting for Akt, downstream proteins, casepase 3, and PARP detection.

In addition to HDAC inhibition, MPT0E028 also possesses a potent, direct Akt targeting ability in a concentration-dependent manner according to the Kinome Diversity Screen Data Report from Ricerca Pharma Services (Taiwan Pharmacol. Lab.) using the enzyme-based ELISA quantitation method (IC_50_ = 5.78 μM) (Fig. 3A). Western blot analysis revealed that MPT0E028 caused a reduction of p-Akt (T308) in Ramos and p-Akt (S473) in BJAB cells, as well as the downstream protein deactivation of p-mTOR and p-GSK3β in a time-dependent manner (Fig. 3B). Total Akt and downstream proteins expressions were also downregulated by MPT0E028 in Ramos cells (Fig. 3B, left panel), whereas these remained unchanged in BJAB cells (Fig. 3B, right panel), suggesting a different modulation of MPT0E028 in different cells. LY294002, a PI3K inhibitor, was used as a positive control to check the basal expression of Akt in both cells. As shown in supplementary Fig. 1,-Akt (S473) and p-Akt (T308) dephoshporylation could only be detected in BJAB cells and in Ramos cells, respectively, indicating that Akt phosphorylation is different among cell lines. To exploit the detailed mechanisms of p-Akt (S473) dephosphorylated by MPT0E028, we focused on BJAB cells. As shown in Fig. 3C, the effect of MPT0E028 on Akt dephosphorylation is about 6 times more potent than SAHA using the cell-based ELISA assay (MPT0E028, IC_50_ = 5.90 ± 1.6 μM; SAHA, IC_50_ = 32.89 ± 11.3 μM). To assess the role of Akt in MPT0E028-induced cell apoptosis, we examined the effect of overexpressing Akt on rescuing MPT0E028-induced apoptotic death by transiently transfect BJAB cells with constitutively active myr-Akt. Transfection of myr-Akt effectively reversed the inhibition effect of MPT0E028 on Akt phosphorylation and its downstream protein activation of p-mTOR and p-GSK3β (Fig. 3D). Moreover, caspase 3 and PARP activation were also abolished when overexpressing Akt in MPT0E028- and SAHA-treated cells (Fig. 3E), confirming that Akt plays a part in MPT0E028-induced cell apoptosis. These results suggest that MPT0E028 induced apoptotic cell death not only by inhibition of HDAC activity but also by directly targeting the Akt-dependent pathway in human B-cell lymphoma cells.

### Correlation between HDAC and Akt inhibition of MPT0E028 on cell apoptosis

**Figure 4:**
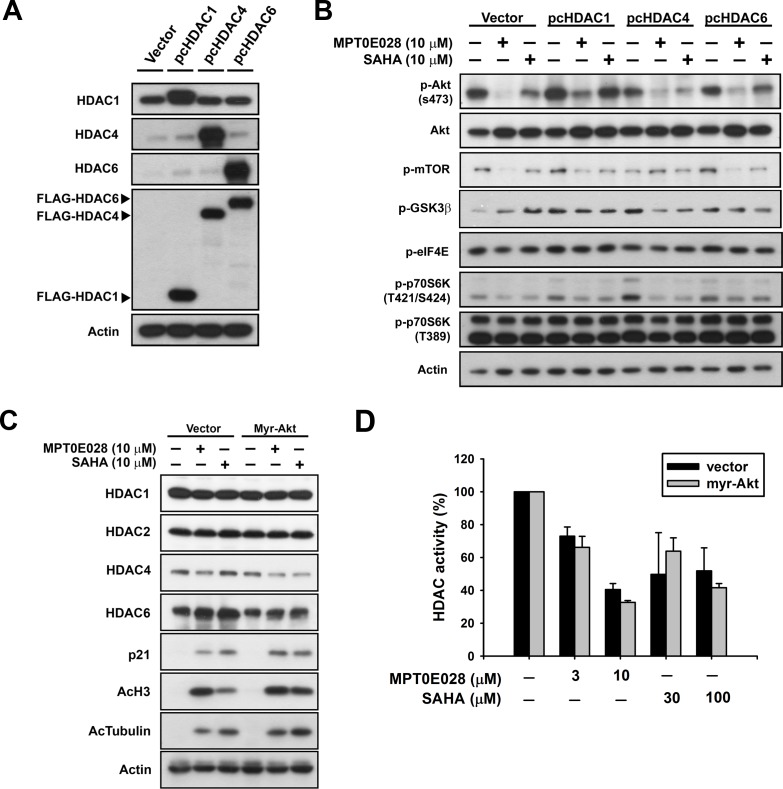
The relationship between HDACs and Akt under the effect of MPT0E028 A. Confirmation of transfection efficiency of HDACs. B. Contribution of HDACs to MPT0E028-inhibited Akt phosphorylation. C. Contribution of Akt to MPT0E028-regulated HDAC protein, marker expression, and D. MPT0E028-inhibited HDAC enzyme activity. BJAB cells were transiently transfected with plasmids encoding vector (pcDNA 3.1), flag-tagged human HDAC1, 4, and 6 or myr-Akt by using nucleofection described in Materials and Methods. Cells were treated with indicated concentration of MPT0E028 or SAHA for 24 h and whole cell lysates were subjected to western blotting for A. HDAC proteins, flag, B. Akt, Akt downstream proteins, C. HDACs and HDAC inhibition marker detection and D. HDAC activities detection as described in Materials and Methods. Data represent mean ± SEM from at least three independent experiments.

It has been reported that HDAC inhibitors may cause Akt depletion through modulation of gene expression [16] and inhibit Akt phosphorylation through disruption of HDAC-PP1 (protein-phosphatase 1) complex [17]. In this study, we aimed to determine the relationship between HDAC and Akt under the inhibitory effect of MPT0E028. First, we transfected BJAB cells with pcHDAC1, pcHDAC4, and pcHDAC6 (Fig. 4A), respectively, to further examine the individual role of HDACs in MPT0E028-inhibited Akt and downstream proteins activation. As shown in Fig. 4B, even though MPT0E028-inhibited p-Akt (S473) was slightly reversed when overexpressing HDAC1 and 6 in BJAB cells, the downstream protein p-mTOR did not reverse in any obvious fashion. The results indicated that MPT0E028 did not inhibit Akt phosphorylation through HDAC inhibition. We then transfected BJAB cells with myr-Akt to examine the role of Akt in rescuing MPT0E028-inhibited HDAC protein expression and enzyme activity. Overexpresssing Akt neither influenced the HDAC protein expression inhibited by MPT0E028 nor the HDAC inhibition markers acetyl-histone 3, acetyl-α-tubulin, or p21 induced by MPT0E028 (Fig. 4C). MPT0E028-inhibited HDAC enzyme activity was similarly unaffected by Akt overexpression (Fig. 4D). These data indicated that MPT0E028 does not reduce HDAC enzyme activity or protein expression through Akt inhibition. Based on these results, it appears that MPT0E028 targets both HDACs and Akt independently.

### MPT0E028 affects many genes and protein expressions

**Figure 5:**
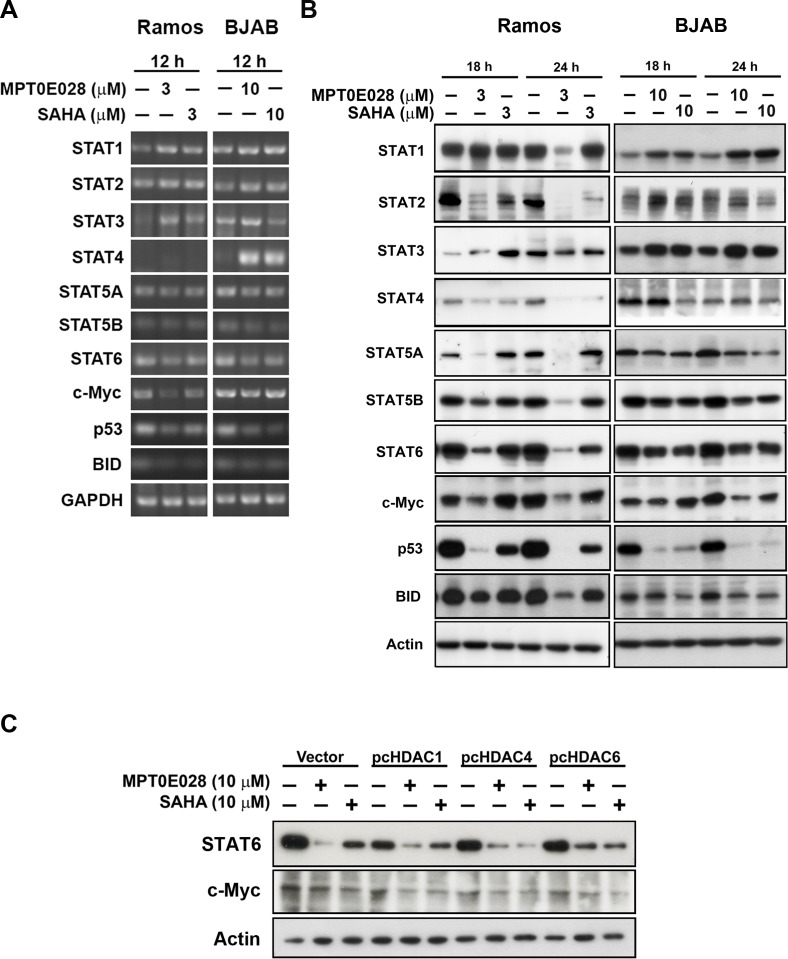
MPT0E028 affect the gene expression in multiple ways in lymphoma cells A. mRNA expression of genes of interest. Cells were treated with MPT0E028 or SAHA for 12 h and then harvest for mRNA detection. B. Protein expression of genes of interest. Cells were treated with MPT0E028 or SAHA for the indicated time interval and then harvested for western blotting detection. C. the role of HDACs in MPT0E028-inhibited STAT6 and c-myc expression. BJAB cells were transiently transfected with plasmids encoding vector (pcDNA 3.1), flag-tagged human HDAC1, 4, and 6 by using nucleofection described in Materials and Methods. Cells were treated with 10 μM MPT0E028 or SAHA for 24 h and whole cell lysates were subjected to western blotting.

Because HDAC inhibitors affect many gene expressions at the transcriptional level, we determined which genes were up- or downregulated by MPT0E028. We extracted RNA from MPT0E028-treated Ramos and BJAB cells and submitted to Phalanx Biotech (Hsinchu, Taiwan). Standard selection criteria to identify differentially expressed genes are established at |Fold change| ≥ 1 and *P* < 0.05. According to the report, around 2,000 genes are upregulated and 1,600 genes downregulated under treatment of MPT0E028. Results showed that MPT0E028 may affect the entire *STAT* family (*STAT1, STAT2, STAT3, STAT4, STAT5A, STAT5B, STAT6*) as well as some oncoproteins such as *MYC, TP53,* and *BID* (Table 1), which are key regulators participating in many pathways. We also performed RT-PCR and western blotting of these genes for mRNA (Fig. 5A) and protein (Fig. 5B) detection. However, analysis of these genes altered in expression revealed cell-line-specific response to MPT0E028. In these genes of interest, we found that *STAT6, TP53*, and *MYC* are inhibited consistently at gene, mRNA, and protein expression in both cell lines (Table 1) (Fig. 5A and 5B). Evidence has shown that some HDACs correlate with STAT6 [18], p53 [19], and c-myc [20]. However, our data show that inhibition of STAT6 and c-myc expression by MPT0E028 may not be mediated by HDACs (HDAC1, 4, and 6) inhibition in B-cell lymphoma cell lines (Fig. 5C). Other genes, however, revealed inconsistent expression of mRNA and protein levels, indicating that complicated regulations may exist at transcriptional and translational levels in the presence of MPT0E028.

**Table 1:** Expressed genes of interest in response to MPT0E028 in Ramos and BJAB cells

			Ramos		BJAB	
Accession #	Gene symbol	Gene name	Fold change	*P*-value	Fold change	*P*-value
NM_007315.3	STAT1	signal transducer and activator of transcription 1, 91kDa	1.66	2.87E-8	1.77	2.57E-12
NM_005419.3^*^	STAT2	signal transducer and activator of transcription 2, 113kDa	1.16	9.46E-8	1.39	0.013
NM_213662.1^§^	STAT3	signal transducer and activator of transcription 3 (acute-phase response factor)	1.71	7.64E-10	1.39	5.40E-10
NM_003151.2	STAT4	signal transducer and activator of transcription 4	N/A	N/A	4.05	1.94E-15
NM_003152.3	STAT5A	signal transducer and activator of transcription 5A	N/A	N/A	−2.15	8.23E-15
NM_012448.3	STAT5B	signal transducer and activator of transcription 5B	N/A	N/A	−1.23	7.61E-8
NR_033659.1^†^	STAT6	signal transducer and activator of transcription 6	−1.57	2.59E-7	−1.71	2.62E-10
NM_002467.4	MYC	v-myc myelocytomatosis viral oncogene homolog (avian)	−3.40	1.68E-28	N/A	N/A
NM_001196.2^¥^	BID	BH3 interacting domain death agonist	−2.19	1.19E-22	−1.78	1.61E-11
NM_000546.4^£^	TP53	tumor protein p53	−2.95	2.78E-16	−2.06	2.17E-14

Ramos and BJAB cells were treated with 3 μM and 10 μM MPT0E028 respectively for 12 h, and then total cellular RNA was extracted for microarray analysis. Each unique splice variant of specific gene was also tested. Different accession numbers are given.

* STAT2: NM_198332.1;

§ STAT3: NM_003150.3, NM_139276.2;

† STAT6: NM_001178079.1, NM_001178080.1, NM_003153.4, NM_001178081.1, NM_001178078.1;

¥ BID: NM_197967.1, NM_197966.1;

£ TP53: NM_001126115.1, NM_001126116.1, NM_001126113.1, NM_001126117.1, NM_001126114.1

### MPT0E028 prolongs survival rate and inhibits tumor growth *in vivo*

**Figure 6:**
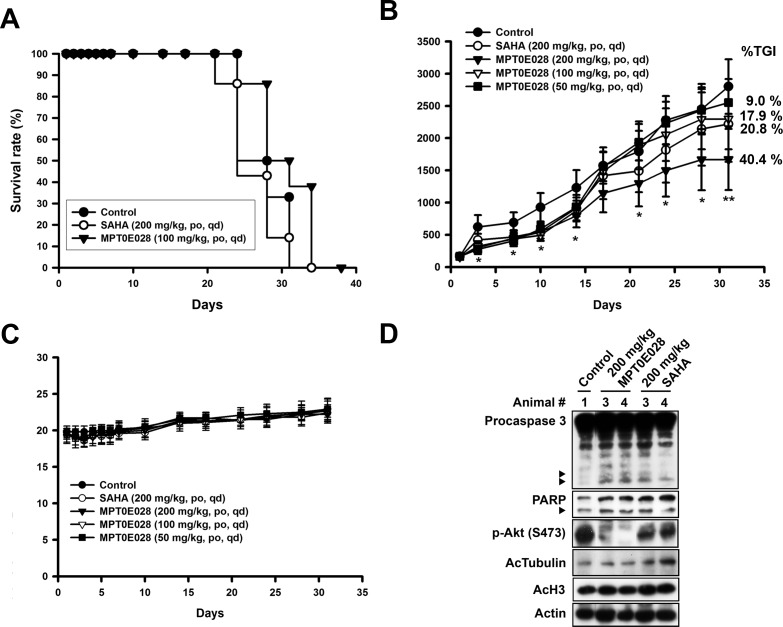
Antitumor effect of MPT0E028 *in vivo* A. MPT0E028-prolonged survival rate in Ramos engrafment model. NOD/SCID mice were engrafted with Ramos cells via tail vein injection. Mice were treated with vehicle, MPT0E028 (100 mg/kg) or SAHA (200 mg/kg) daily by oral gavage. B. MPT0E028-inhibited tumor growth in nude mice bearing the BJAB tumors. Nude mice were treated with vehicle or 50-200 mg/kg of MPT0E028 or 200 mg/kg of SAHA daily by oral gavage for 31 days. Seven mice per group were used in the xenograft experiment (*P < 0.05; **P < 0.01). C. Body weight of the BJAB tumor bearing mice under the treatment during the study. Data represent mean ± SEM from seven mice in each group. D. BJAB xenograft tumors were subjected to western analysis for caspase 3, PARP, p-Akt (S473), acetyl-histone 3, and acetyl-α-tubulin detection.

On the basis of MPT0E028-induced apoptotic effect *in vitro*, we further examined the *in vivo* antitumor effect of MPT0E028 in two animal models. We produced a Ramos engrafment in NOD/SCID mice and a BJAB xenograft in nude mice. In the Ramos engrafment model, mice were randomly divided into three groups receiving the following daily treatment by oral gavage: (a) vehicle, (b) MPT0E028 100 mg/kg, and (c) SAHA 200 mg/kg. The end point used for this study was survival rate. Survival rate in the MPT0E028-treated group was significantly prolonged compared with the control group, whereas the SAHA-treated group showed no significant efficacy (Fig. 6A). In the BJAB xenograft model, mice were randomly divided into five groups receiving the following daily treatment, also by oral gavage: (a) vehicle, (b) MPT0E028 50 mg/kg, (c) MPT0E028 100 mg/kg, (d) MPT0E028 200 mg/kg, and (e) SAHA 200 mg/kg. MPT0E028 showed an effective tumor reduction effect in a dose-dependent manner (40.4% of TGI, total growth inhibition) (Fig. 6B). The 200 mg/kg SAHA-treated group showed an effect similar to the 100 mg/kg MPT0E028-treated group (17.9% and 20.8% of TGI, respectively), representing more potent tumor reduction than MPT0E028. We also observed no significant difference in body weight between these groups, indicating that MPT0E028 exhibits little apparent toxicity *in vivo* (Fig. 6C). After 31 days of treatment, the mice were sacrificed, and tumors were carefully removed for western blot analysis. As shown in Fig. 6D, caspase 3 and PARP were activated in MPT0E028-treated and SAHA-treated groups, with MPT0E028 inducing a more profound effect. The acetylation levels of histone 3 and -tubulin were also increased in MPT0E028- and SAHA-treated groups, and MPT0E028-inhibited Akt phosphorylation was also detected. As our data showed, the effect of MPT0E028 *in vivo* is consistent with that *in vitro*. Taken together, the antitumor activity of MPT0E028 against human B-cell lymphoma tumor, as demonstrated by both engrafment and xenograft, was superior to that of SAHA, proving MPT0E028's potent antitumor effect *in vivo*.

## DISCUSSION

Epigenetic dysregulation occurs in many malignant cells, possibly due to histone modification leading to chromatin remodeling and abnormal gene expression. Histones can be modified in many different ways, including acetylation, methylation, phosphorylation, sumoylation and ubiquitination [21]. Modifying epigenetic changes is a recognized therapeutic strategy, and histone acetylation is one of these approaches. HDAC inhibitors relax the condensed chromatins, allowing the re-expression of genes that control cell proliferation and survival. On the other hand, HDAC inhibitors may cause non-histone substrates acetylation, which have been implicated in the anticancer activity of HDAC inhibitors [22, 23]. Many HDAC inhibitors are in clinical trials and have become promising agents in recent years. We previously demonstrated that MPT0E028, a novel HDAC inhibitor, exhibited potent anticancer ability toward various cell lines, including hematological malignances and solid tumors. This revealed a forceful apoptosis-inducing and HDAC inhibitory effect on colorectal carcinoma *in vitro* and *in vivo* [12]. Since current HDAC inhibitors are more effective against certain hematological malignancies such as cutaneous T-cell lymphoma (CTCL) [24], but not B-cell lymphoma, we further investigated the antitumor effect and detail mechanisms of MPT0E028 in B-cell lymphomas *in vitro* and *in vivo*. Our results demonstrate that MPT0E028 targeted both Akt and HDACs while inducing B-cell lymphoma cell apoptosis, suggesting MPT0E028 as a novel antitumor agent with dual functionality.

Combining HDAC inhibitors and PI3K/Akt pathway inhibition has been shown to further induce apoptosis in human cancer cells *in vitro* and *in vivo* [25]. For example, combining HDAC inhibitors and LY294002 (a PI3K inhibitor) sensitizes a non-small cell lung cancer (NSCLC) xenograft to apoptosis [26]; in prostate cancer, HDAC inhibitor produces greater antitumor activity when combines with PI3K/mTOR inhibitor [27]. Co-administration of HDAC inhibitors with perifosine (an Akt inhibitor) and rapamycin (an mTOR inhibitor) in human leukemia cells also promotes mitochondria injury and apoptosis [28, 29]. Because MPT0E028 targets both HDACs and the Akt pathway, the benefit of omission from combination treatment and efficacious outcomes are predictable. Combination treatment with HDAC inhibitors plus PI3K/Akt pathway inhibition also potentiates the targeting effect of tumor angiogenesis [30], invasion, and migration [31], indicating MPT0E028's wide-ranging usefulness. However, detailed function and mechanisms need to be further investigated.

Many studies have demonstrated that HDAC inhibitors also influence Akt phosphorylation [32, 33], but few studies have identified the relationship between HDAC and Akt under the effect of a HDAC inhibitor. Evidence show that HDAC inhibitors, such as valproic acid and butyrate, impede Akt1 and Akt2 expression, which leads to Akt deactivation and apoptotic cell death [16]. In another study, Chen *et al*. demonstrated that HDAC inhibitors cause Akt dephosphorylation by disrupting HDAC-protein phosphatase 1 (PP1) complexes, with HDAC1 and 6 contributing to this effect much more [17]. Consistent with our data, we show that overexpression of HDAC1potentiates the Akt-phopshorylation level at the baseline (Fig. 4B), and the MPT0E028-inhibited Akt phosphorylation could be partially rescued when HDAC1 and 6 were overexpressed. However, in this study, the Akt downstream protein activation (e.g., p-mTOR, p-GSK3β, p-p70S6K and p-eIF4E) inhibited by MPT0E028 apparently could not be rescued when HDACs were overexpressed, suggesting that function was not fully recovered under the inhibition effect of MPT0E028 (Fig. 4B). Besides, overexpressing Akt did not affect HDAC expression (Fig. 4C) or enzyme activity (Fig. 4D), demonstrating that MPT0E028 inhibits HDAC activity parallel to Akt deactivation. In Ramos cells, total Akt protein expression was depleted under the effect of MPT0E028 (Fig. 3B, left panel). One possible mechanism may involve transcription inhibition [16], another possible explanation may relate to Hsp90 and proteasome degradation. Evidence has shown that HDAC6 directly regulates Hsp90 acetylation, and Akt is a client protein of Hsp90; therefore, inhibition of HDAC6 may disrupt the chaperone function of Hsp90, leading to Akt depletion [34, 35]. We also performed microarray analysis in Ramos cells and observed that *Akt1* was downregulated (Log2 ratio = −1.082, ***P value < 0.001) under treatment of MPT0E028 (data not shown), suggesting MPT0E028 may influence Akt protein expression through transcription inhibition. Thus, MPT0E028 may inhibit Akt activation through different mechanisms due to characteristics of different cell types.

The values of IC_50_ obtained from our enzyme-based HDAC activity assay [12] were around 100 times lower than that obtained from our cell-based assay (Fig. 2A). This phenomenon may be explained by the existence of a much more complicated environment in whole cells that prevents MPT0E028 from achieving its targets. However, enzyme-based (Fig. 3A) and cell-based Akt activity assay (Fig. 3C) show controversially similar values of IC_50_, both around 5 μM, illustrating other indirect mechanisms may exist that facilitate the Akt-deactivation effect of MPT0E028. Chen *et al*. demonstrated that HDAC inhibitors cause Akt dephosphorylation by disrupting HDAC-protein phosphatase 1 (PP1) complexes [17], thereby releasing PP1 to deactivate Akt. MPT0E028 treatment in whole cells may not only target Akt directly but also inhibit phosphorylation through HDAC inhibition indirectly, therefore facilitating MPT0E028-inhibited Akt phosphorylation.

Since HDAC inhibitors modify histone acetylation, many genes may turn up or down due to epigenetic modulation under treatment [36, 37]. Microarray analysis showed that MPT0E028 may influence many genes, including the *STATs* family and some oncoproteins, in B-cell lymphoma cells (Table 1). We found that STAT6, p53, and c-myc are consistent in gene, mRNA, and protein expression in both cells (Fig. 5A and 5B). STAT6 has been found to be constitutively active in some leukemia and lymphoma types, associated with cell proliferation and transformation [38]. SAHA may inhibit STAT6 mRNA and protein expression therefore decrease cell proliferation [38]. In our study, STAT6 was profoundly inhibited at transcriptional and translational levels under treatment of MPT0E028 (Fig. 5A and 5B). However, we found that HDAC1, 4, and 6 contributed little to MPT0E028-inhibited STAT6 expression (Fig. 5C), indicating that MPT0E028 may regulate STAT6 expression in a HDACs-independent manner. HDAC inhibitors that cause mutant p53 degradation by either restoring or mimicking p53 trans-functions [39] or through HDAC6-Hsp90 chaperone axis have been reported [40]. Because Ramos (p53^mutant^ 1254D) and BJAB (p53^mutant^ H193R) cells both harbor mutant p53, MPT0E028-caused p53 degradation may be expected. However, p53 mRNA could also be inhibited by MPT0E028 through unknown mechanisms (Fig. 5A). *MYC*, one of the major oncogenes in B-cell lymphoma [41], has also been affected by MPT0E028 (Fig. 5A and 5B). Evidence shows that HDAC-mediated deacetylation alters the transcriptional activity of nuclear transcription factors such as c-myc [42]. In our study, we found that HDAC1, 4, and 6 are barely evident in MPT0E028-inhibited c-myc expression (Fig. 5C). Recent findings show that HDAC3 may cooperate with c-myc to regulate microRNA expression and therefore malignant transformation in aggressive B-cell malignancies [20]. MPT0E028 may probably inhibit these cellular proteins through unknown mechanisms that need to be further elucidated. However, MPT0E028 at concentrations causing apoptosis decreases the expression of STAT6 and c-myc. Thus, downregulation of STAT6 and c-myc may play a part in MPT0E028-induced apoptosis in B-cell lymphoma.

Clinical trials using HDAC inhibitors alone have shown limited efficacy against solid tumors. Therefore, combinations with classical chemotherapeutic agents [43, 44] or new small molecule inhibitors [45] are being investigated. According to our previous study, MPT0E028 also showed synergism in combination with tyrosine kinase inhibitors, such as erlotinib and sorafenib, in non-small cell lung cancer (NSCLC) and liver cancer, respectively [13, 14]. Therefore, combination treatment with different therapeutic agents in solid tumors may broaden the potential usage of MPT0E028. In the case of B-cell lymphoma, HDAC inhibitor has shown synergistic effect with anti-CD20 antibody rituximab [46]. However, emerging evidence has demonstrated that rationale should be followed to combine HDAC inhibitors with immunotherapy to obtain synergistic effect due to complexity of HDAC inhibitors on immune system and tumor microenvironment [47]. Although MPT0E028 shows significant antitumor effect in B-cell lymphoma, detailed mechanisms still need to be further investigated.

In conclusion, our data suggest that MPT0E028 is a promising and effective anti-cancer HDAC inhibitor. We demonstrated that MPT0E028 exhibits a potent dual function of HDAC and Akt inhibition, leading to B-cell lymphoma apoptosis *in vitro* and *in vivo*. Its antitumor activities include inducing apoptosis, HDAC activity inhibition, Akt pathway deactivation, and regulation of many genes *in vitro*; and prolongation of survival rate and reduction of tumor volume *in vivo*. Our previous work [12-14] and current studies suggest that MPT0E028 has greater efficacy *in vivo* and *in vitro* compared to SAHA. In totality, our results provide compelling evidence that MPT0E028 can be an effective antitumor agent in the treatment of B-cell lymphoma.

## MATERIALS AND METHODS

### Reagents

MPT0E028 and SAHA were synthesized by Professor Jing-Ping Liou. RPMI-1640 medium, FBS, penicillin, streptomycin, and all other tissue culture reagents were obtained from GIBCO/BRL Life Technologies (Grand Island, NY, USA). 3-(4,5-Dimethylthiazol-2-yl)-2,5-diphenyltetrazolium bromide (MTT), propidium iodide (PI), LY294002 and all of the other chemical reagents were purchased from Sigma Chemical (St. Louis, MO, USA). The following antibodies were used: caspase 8, caspase 9, p-Akt(T308), Akt, p-mTOR, mTOR, p-GSK3β, p-eIF4E, p-p70S6K(T421/S424), p-p70S6K(T389), HDAC1, HDAC2, HDAC4, acetyl-α-tubulin, BID, STAT2, STAT4, STAT5A, STAT6 (Cell Signaling Technologies, Boston, MA, USA); PARP, HDAC6, HRP-conjugated anti-mouse and anti-rabbit IgG (Santa Cruz Biotechnology, Santa Cruz, CA, USA); p-Akt (S473) (Epitomics, Burlingame, CA, USA); caspase 6, caspase 7, p53, STAT1 (BD Biosciences PharMingen, San Jose, CA, USA); caspase 3 (Imgenex, San diego, CA, USA); acetyl-histone H3, STAT5B (Upstate Biotechnology, Lake Placid, NY, USA); Actin (Chemicon, Billerica, MA, USA). Trizol reagent was from Invitrogen (Carlsbad, CA, USA). Random primer and M-MLRT were purchased from Promega (Madison, WI, USA). Pro-Teq was from Protech (Taipei, Taiwan).

### Cell culture

Two fast-growing malignant B cell lymphoma (Burkitt lymphoma) cell lines and human umbilical vein endothelial cells (HUVEC) were used. Ramos and HUVEC cells were purchased from American Type Culture Collection (Manassas, VA, USA) and BJAB cells were a kind gift from Professor Ping-Ning Hsu (National Taiwan University Hospital and National Taiwan University, College of Medicine, Taipei, Taiwan). Ramos and BJAB cells were cultured in RPMI-1640 with 10% heat-inactivated fetal bovine serum and penicillin (100 units/ml)/streptomycin (100 μg/ml) while HUVEC was cultured in M199 with 20% heat-inactivated fetal bovine serum (v/v) and penicillin (100 units/mL)/streptomycin (100 μg/mL). All cells were maintained in humidified air containing 5% CO_2_ at 37°C. Cell density was maintained between 1×10^5^ and 1×10^6^ cell/ml and passaged every 2 to 3 days.

### MTT assay

Cell viability was assessed by MTT assay. Ramos and BJAB cells were plated in 24-well plate (4×10^5^ cells/well) while HUVEC cells were plated in 96-well plate (5000 cells/well) and treated with different doses of MPT0E028 and SAHA for 24 h. After the treatment, 100 μl of 0.5 mg/ml 3-(4,5-Dimethylthiazol-2-yl)-2,5-diphenyl-tetrazolium bromide (MTT) were added to each well and incubated at 37°C to stained cells forming an insoluble blue formazan product. After 1 h incubation, 100 μl of extraction reagent (0.1M sodium acetate buffer or DMSO) were added to each well and mix thoroughly to lyse cells. The plates were then measured at 550 nm using an enzyme-linked immunosorbent assay (ELISA) reader (Packard, Meriden, CT, USA).

### Protein extraction and western blot

After treatment of cells for the indicated agents and time course, cells were washed with ice-cold PBS and lysed with ice-cold lysis buffer (1 mM EGTA, 1 mM EDTA, 150 mM NaCl, 1% Triton X-100, 2.5 mM sodium pyrophosphate, 1 mM PMSF, 1 mM Na_3_VO_4_, 1 μg/ml leupeptin, 1 μg/ml aprotinin, 5 mM NaF in 20 mM Tris–HCl buffer, pH 7.5) to extract protein. Equivalent aliquots of protein were electrophoresized on 8 to 15% sodium dodecyl sulfate-polyacrylamide gel electrophoresis and transferred to poly(vinylidene difluoride) membranes. The membranes were incubated with specific antibodies overnight at 4°C and then applied to appropriate horseradish peroxidase-conjugated anti-mouse or anti-rabbit immunoglobulin G secondary antibodies for 1 h at room temperature. Signal detection was performed with chemiluminescence reagents (Amersham, Buckinghamshire, UK).

### Flow cytometry analysis

Following drug treatment, cells were harvested and washed with PBS, and then the pellets were resuspended and fixed in ethanol (75%, v/v) overnight at −20°C. After centrifugation, the fixed cells were washed with ice-cold PBS once and incubated in 0.1M of phosphate–citric acid buffer (0.2 M NaHPO_4_, 0.1 M citric acid, pH 7.8) for 30 min at room temperature. The cells were centrifuged and stained with propidium iodide staining buffer containing Triton X-100 (0.1%, v/v), RNase A (100 mg/ml) and propidium iodide (80 mg/ml) for 30 min. Cell cycle distribution was performed using a FACScan flow cytometry with CellQuest software (Becton Dickinson, Mountain View, CA, USA).

### ELISA for Akt detection

BJAB cells were plated in 6-cm dish (1×10^6^ cells/ml) and treated with different doses of MPT0E028 and SAHA for 24 h. After the treatment, cells were lysed with ice-cold lysis buffer mentioned above and equivalent aliquots of protein were subjected to a PathScan phospho-Akt1 (Ser473) sandwich ELISA kit (Cell Signaling Technology) following manufacture's instruction. In briefly, whole cell lysate was first half-diluted with sample diluent and added 100 μl into the well incubating overnight at 4°C. After the reaction, wells were washed 4 times with 200 μl wash buffer and then added 100 μl of Akt1 mouse detection antibody incubating 1 h at 37°C. Washed 4 times again and then added 100 μl of anti-mouse IgG HRP-linked antibody incubating 30 min at 37°C. After incubation, washed the well once again and then added 100 μl TMB substrate for color production and finally added 100 μl STOP solution. The results were measured by spectrophotometer at wavelength 450 nm.

### Akt kinase activity

MPT0E028 was submitted to MDS Pharma Services (Taiwan Ltd) for Kinome Diversity Screening using enzyme-based kinase activity assay. In briefly, Akt activity was assayed using human recombinant insect Sf21 cells for Akt kinase source and crosstide peptide (GRPRTSSFAEG, 15 μg/mL) as a substrate. Cell lysate was incubated with kinase assay buffer (50 mM HEPES pH 7.4, 10 mM MgCl_2_, 0.2 mM Na_3_VO_4_, and 1 mM DTT) for 60 min at 25°C, and analyzed by ELISA quantitation of Crosstide-P.

### Cell-based HDAC fluorescence activity assay

Cells were plated in 10-cm dish (1×10^6^ cells/ml) and treated with different doses of MPT0E028 and SAHA for 24 h, and then cell lysate was subjected to a HDAC Fluorometric Activity Assay Kit (K330-100, Biovision Inc.) to determine the HDAC activity. 50 μg of cell lysate was added to react with fluorometric substrate t-butoxycarbonyl-Lys (AC)-amido-4-methylcoumarin (Boc-Lys (AC) AMC) and incubated at 37°C for 30 minutes. After the incubation, the lysine developer in this kit was added and the mixture was incubated for another 30 min at 37°C. Fluorescence was detected by plate reader paradigm detection platform (Beckman coulter) with Ex=360 (excitation) and Em=465 nm (emission).

### Transient transfection

Myr-Akt plasmids were kindly provided by Professor Chien-Huang Lin (Taipei Medical University, Taipei, Taiwan). PcDNA-FLAG-HDAC1 (plasmid 13820), pcDNA-FLAG-HDAC4 (plasmid 13821), pcDNA-FLAG-HDAC6 (plasmid 13823) were purchased from Addgene (Cambridge, MA, http://www.addgene.org). Transfections for BJAB cells were done by nucleofection using nucleofector solution L, program T-016, according to the manufacturer's instructions (Amaxa, Inc.). A total of 4×10^6^ cells were used per transfection and mixed with 0.5 or 1 μg of myr-Akt, pcHDAC1, pcHDAC4 and pcHDAC6. After transfection, BJAB cells were recovered for 24 h incubating at 37°C.

### RT-PCR

For RT-PCR analysis, total cellular RNA was extracted from BJAB cells using Trizol reagent (Invitrogen Corp., Carlsbad, CA, USA) following the manufacturer's instruction. First strand cDNA was synthesized using 5 μg of mRNA incubating with random primer at 65°C for 5 min and then reacting with moloney murine leukemia virus reverse transcriptase (M-MLVRT) at 37°C for 1 h. PCR was then performed with primers for STAT1(5′-AAGGTGGCAGGATGTCTCGT-3′/5′-TGGTCTCGTG

TTCTTCTGTTCTG-3′), STAT2 (5′-AAGCACTGCTAGGCCGATTA-3′/5′-GGCT

GGGTTTCTACCACAAA-3′), STAT3 (5′-TGGAAGAGGCGGCAGCAGATAGC

-3′/5′-CACGGCCCCCATTCCCACAT-3′), STAT4 (5′-TCAAGTCCAACAGTTAGAAATCA-3′/5′-TAAACAGTTTGAAATAACCACAG-3′), STAT5A (5′-CACAGATCAAGCAAGTGGTC-3′/5′-CTGTCCATTGGTCGGCGTAA-3′), STAT5B (5′-G

TAAACCATGGCTGTGTGGA-3′/5′-AAATAATGCCGCACCTCAAT-3′), STAT6 (5′-CCAAGACAACAACGCCAAAGC-3′/5′-AGGACACCATCAAACCACTGCC-3′), p53 (5′-CAGCCAAGTCTGTGACTTGCACGTAC-3′/5′-CTATGTCGAAAAGTGTTTCTGTCATC-3′), c-myc (5′-TACCCTCTCAACGACAGCAG TCTTGACAT

TCTCCTCGGTG-3′) and done with following the procedure: after a “hot-start” for 5-10 min at 94°C, 25-35 cycles were used for amplification, with a melting temperature of 94°C, an annealing temperature of 53-68°C, and an extending temperature of 72°C, each for 1 min, followed by a final extension at 72°C for 7 min. PCR products were separated on 1.5% agarose gel and visualized by ethidium bromide staining.

### Microarray analysis

Ramos and BJAB cells were treated with 3 μM and 10 μM MPT0E028 for 12 h respectively, and then total cellular RNA was extracted using Trizol reagent (Invitrogen Corp., Carlsbad, CA, USA) following the manufacturer's instruction. Total cellular RNA was purified using the Purelink™ HiPure Plasmid Midiprep kit (Invitrogen, Carlsbad, CA). Purified RNA samples were submitted to Phalanx Biotech (Hsinchu, Taiwan) for microarray analysis. We used Phalanx Human OneArray to measure MPT0E028-induced gene expression and detailed description of Phalanx Biotech company microarray procedure can be found at http://www.phalanxbiotech.com/. Each microarray contains 30,275 oligonucleotides: 29,187 human genome probes, and 1,088 experimental control probes. Each array was performed in duplicate. The genes with a threshold of 1.2 times fold-change were selected and BioCarta Pathway and KEGG (Kyoto Encyclopedia of Genes and Genomes) pathway databases were used to identify functionally related gene pathways.

### *In vivo* Ramos engraftment model and BJAB xenograft model

Male NOD/SCID mice and nude mice (NTUH Animal Facility) were 5 weeks old and had a body weight (BW) range of 20-24 g on day one of the study. The mice were carefully maintained and all animal procedures were in accordance with the Institutional Animal Care and Use Committee procedures and guidelines. All animal experiments followed ethical standards, and protocols has been reviewed and approved by Animal Use and Management Committee of National Taiwan University (IACUC Approval No: 20100225).

In Ramos engrafment model, human B-cell lymphoma Ramos cells were resuspended at 10^7^ cells/ml in culture medium at room temperature, and 2×10^6^ cells were inoculated into male NOD/SCID mice via tail vein. Treatment began 1 week after engrafment. Both MPT0E028 and SAHA were dissolved in vehicle (0.5% carboxymethyl cellulose + 0.1 % Tween 80 in 5% dextrose). After engrafment, mice were randomly placed into three groups and received the following treatment by oral gavage during the study: (a) vehicle alone, (b) MPT0E028 at 100 mg/kg daily, and (c) SAHA at 200 mg/kg daily. Survival rate was used as end point for this study.

In BJAB xenograft model, the male nude mice were injected subcutaneously with the same volume of BD Matrigel Matrix HC (BD bioscience, catalog 354248) and BJAB cells (2×10^7^ cell/mouse) into the flank of each animal. When the tumors had grown to the size of around 80 to 100 mm^3^, the animals were divided into five groups (n=7) and receive the following treatment by oral gavage for 31 days during the study: (a) vehicle alone, (b) MPT0E028 at 50 mg/kg daily, (c) MPT0E028 at 100 mg/kg daily, (d) MPT0E028 at 200 mg/kg daily, and (e) SAHA at 200 mg/kg daily. Both MPT0E028 and SAHA were dissolved in vehicle (0.5% carboxymethyl cellulose + 0.1 % Tween 80 in 5% dextrose). Tumor size was measured twice weekly and calculated from V = 0.5 lw^2^, where w = width (w) and l = length (l). At the end of study, tumors were carefully removed and frozen in liquid nitrogen for subsequent western analysis.

### Statistical analysis

Each experiment was performed at least three times and the data are presented as mean ± SEM for the indicated number of separate experiments. Student's *t*-test was used to compare the mean of each group with that of the control group in experiments and one-way ANOVA was used in animal study. *P*-values less than 0.05 were considered significant (*P<0.05, **P<0.01, ***P<0.001).

## SUPPLEMENTARY MATERIAL FIGURE


