# Supplementary material for: Novel oral histone deacetylase inhibitor, MPT0E028, displays potent growth-inhibitory activity against human B-cell lymphoma in vitro and in vivo
Source: Oncotarget. 2014 Dec 31;6(7):4976–91. doi: 10.18632/oncotarget.3213 (PMC4467128; doi:10.18632/oncotarget.3213)
Supplement: Supplementary file 1 [file oncotarget-06-4976-s001.pdf]

Novel oral histone deacetylase inhibitor, MPT0E028, displays potent growth-inhibitory activity against human B-cell lymphoma *in vitro* and *in vivo*

Supplementary Material

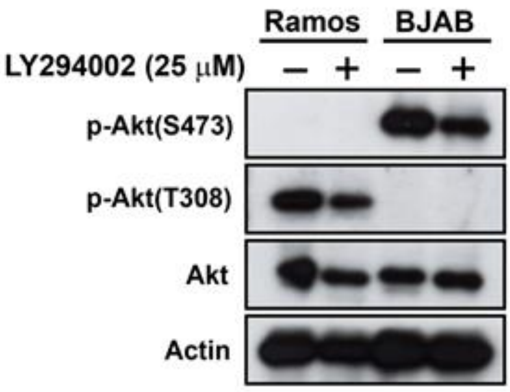

Supplemental Figure 1: Basal expression of p-Akt in Ramos and BJAB cells.

Both cells were treated with 25 μM of LY294002 for 24 h and harvested for p-Akt (S473), p-Akt (T308), and Akt detection using western blotting.
